# A novel puncture approach via point “O” for percutaneous kyphoplasty in patients with L4 or L5 osteoporotic vertebral compression fracture

**DOI:** 10.1038/s41598-022-23732-6

**Published:** 2022-11-07

**Authors:** Jiahu Huang, Jin Yang, Lanjing Chen, Yinzhi Xu, Song Wang

**Affiliations:** 1 Department of Orthopaedics, The Affiliated Hospital of Southwest Medical University, Luzhou, China; 2 Department of Imaging, The Affiliated Hospital of Southwest Medical University, Luzhou, China

**Keywords:** Outcomes research, Fracture repair

## Abstract

This study introduced a novel point “O” puncture approach for percutaneous kyphoplasty (PKP) in patients with L4 or L5 osteoporotic vertebral compression fracture (OVCF) and evaluated its clinical and radiographic outcomes. Between September 2019 and December 2020, we compared the clinical and radiographic outcomes in 31 cases (36 vertebrae) using the “O” entry point PKP intervention (O-PKP) and 31 cases (37 vertebrae) using transverse the process-pedicle approach PKP intervention (T-PKP). No serious postoperative complications were observed in any of the participants. Only two T-PKP patients experienced intervertebral disc space leakage. Compared with the T-PKP patients, the O-PKP patients showed shorter operative time and fluoroscopy times (*P* < 0.05), comparable blood loss and cement volume (*P* > *0.05*), improved VAS and ODI scores at the final follow-up (*P* < 0.05), better increases in the vertebral compression ratio (*P* < 0.05), comparable Cobb angle (*P* > 0.05), comparable anteroposterior bone cement distribution, enhanced bilateral bone cement distribution (*P* < 0.05), and larger sagittal and transverse angles (*P* < 0.05). Herein, O-PKP was indicated for patients with L4 or L5 OVCF. This puncture approach showed significant advantages over T-PKP not only in terms of pain relief, surgery and fluoroscopy times but also in the puncture angle, vertebral reconstruction, and symmetrical cement distribution.

## Introduction

Percutaneous vertebroplasty was first reported as a treatment for aggressive vertebral hemangioma by Galibert et al. in 1987^1^. By the beginning of the twenty-first century, balloon kyphoplasty was applied in clinical settings^2^. Compared to percutaneous vertebroplasty, percutaneous kyphoplasty (PKP) has the advantages of better restoration of the vertebral height and reduction of the spinal kyphosis deformity while minimizing bone cement leakage^3^. In 2004, the use of a uni-pedicular approach for balloon kyphoplasty was proposed by Hoh et al^4^. Compared with bilateral PKP, unilateral PKP offers a shorter operative time, lower radiation exposure, and reduced costs^5^. Researchers have attempted to improve puncture approaches to improve both clinical outcomes and bone cement distribution. Hence, at the present time, there are multiple unilateral puncture approaches, including the conventional transpedicular approach (CPA), transverse process-pedicle approach (TPA), and the extrapedicular approach^6^. These puncture approaches are considered both safe and effective^7,8^. However, to date, most of these studies have analyzed puncture of the thoracic and thoracolumbar vertebrae with only small numbers of lower lumbar vertebrae. Therefore, issues related to lower lumbar kyphoplasty have received relatively little attention.

Our previous clinical research showed that it is, indeed, difficult for these unilateral puncture approaches to reach the midline of the L4 and L5 vertebral bodies to achieve a satisfactory bone cement distribution. A bilateral puncture approach, therefore, may be a viable option for L4 or L5 OVCF. Nevertheless, this method is also associated with various disadvantages, as previously described^5^. Given these challenges, in this study, we preferred to use the unilateral puncture approach. In a previous study^9^, we observed that, compared with CPA, TPA has a more lateral puncture entry point, a wider and safer range of the puncture transverse angle, and a higher success rate. The extrapedicular approach resulted in a lower risk of entry into the intraspinal compartment, compared with CPA and TPA, allowing better entry into the vertebral midline as the entry point of the extrapedicular approach is more lateral than CPA and TPA^10^. However, due to iliac shielding and the width of the L4 and L5 vertebrae, it is very difficult to apply these approaches in these regions^6,9^. Thus, we developed a novel extrapedicular approach using the entry point “O” to perform the PKP for L4 or L5 OVCF. Here, we proposed a novel unilateral puncture approach that avoids iliac shielding, produces large sagittal and transverse angles, achieves symmetric bone cement distribution, and resulted in appropriate clinical effectiveness.

## Clinical data and methods

### Patients

Between June 2020 and December 2020, a total of 31 patients (36 vertebrae) with L4 or L5 OVCF presented at our department and all of them were treated with PKP via the entry point “O” (O-PKP group). To compare the preliminary clinical and imaging outcomes between PKP via the entry point “O” and PKP via TPA, another 31 patients (37 vertebrae) with L4 or L5 OVCF were enrolled consecutively from September 2019 to May 2020, all of them were treated with PKP via TPA (T-PKP group).

The inclusion criteria included: (i) L4 or L5 osteoporotic vertebral compression fracture; (ii) severe low back pain; (iii) osteoporosis diagnosis, based on the World Health Organization criteria (T-Score ≤ − 2.5); (iv) an acute or subacute OVCF on MRI; and (v) patients who can tolerate the surgery. The exclusion criteria included: (i) diagnosis of non-osteoporotic body fractures (including spinal tuberculosis, tumor, and infection); and (ii) vertebral fracture with spinal cord or nerve injury.

All patients signed informed consent before surgery, and they completely understood the risks involved with the surgery, complications, and procedure. The study also received ethical approval from the Ethics Committee of the Affiliated Hospital of Southwest Medical University, and strictly followed the guidelines of the Declaration of Helsinki.

### Surgical intervention

All patients were operated in the prone position under local anesthesia. The fractured vertebral body was located via C-arm fluoroscopy.

**Figure 1:**
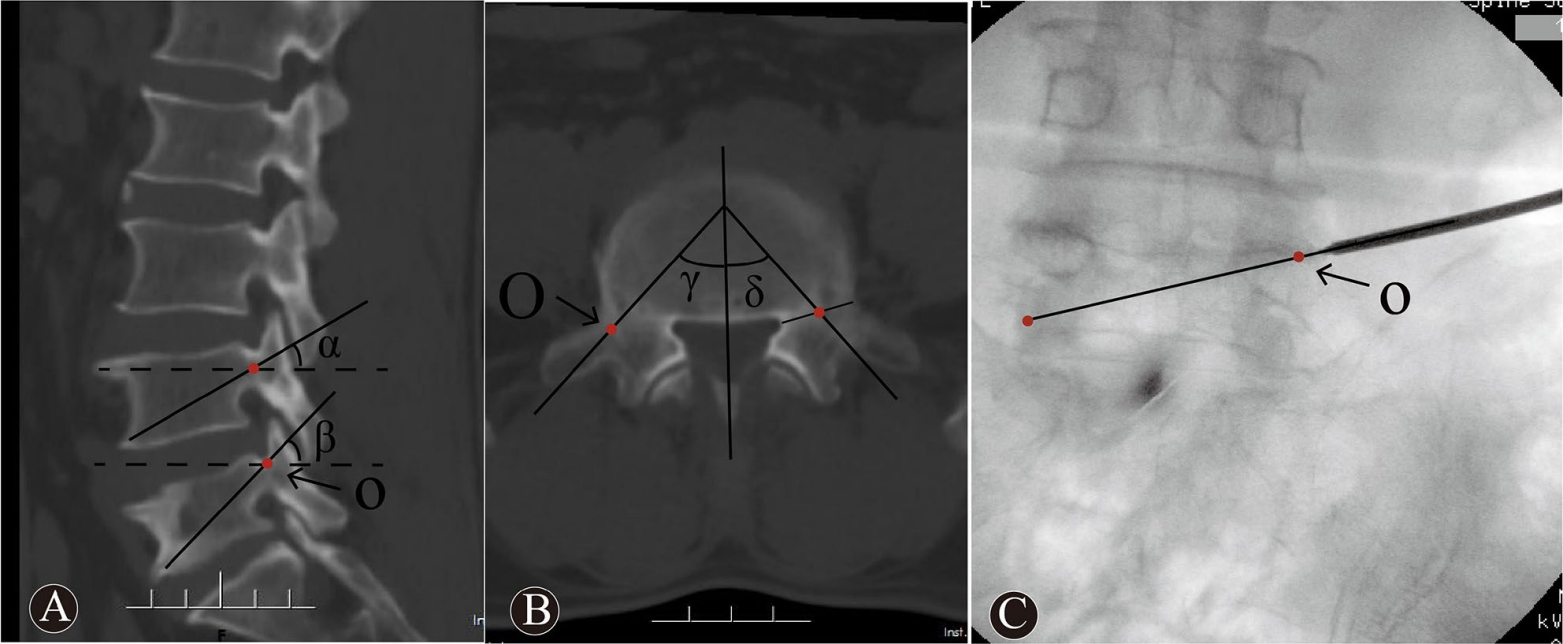
Images showed the puncture approach via point “O” and TPA. The sagittal CT image (**A**) was showed that α and β were sagittal puncture angle of T-PKP and O-PKP, respectively. The cross-sectional CT image (**B**) was showed that γ and δ were transverse puncture angle of O-PKP and T-PKP, respectively. The X-ray image (**C**) was showed the puncture approach of O-PKP.

**Figure 2:**
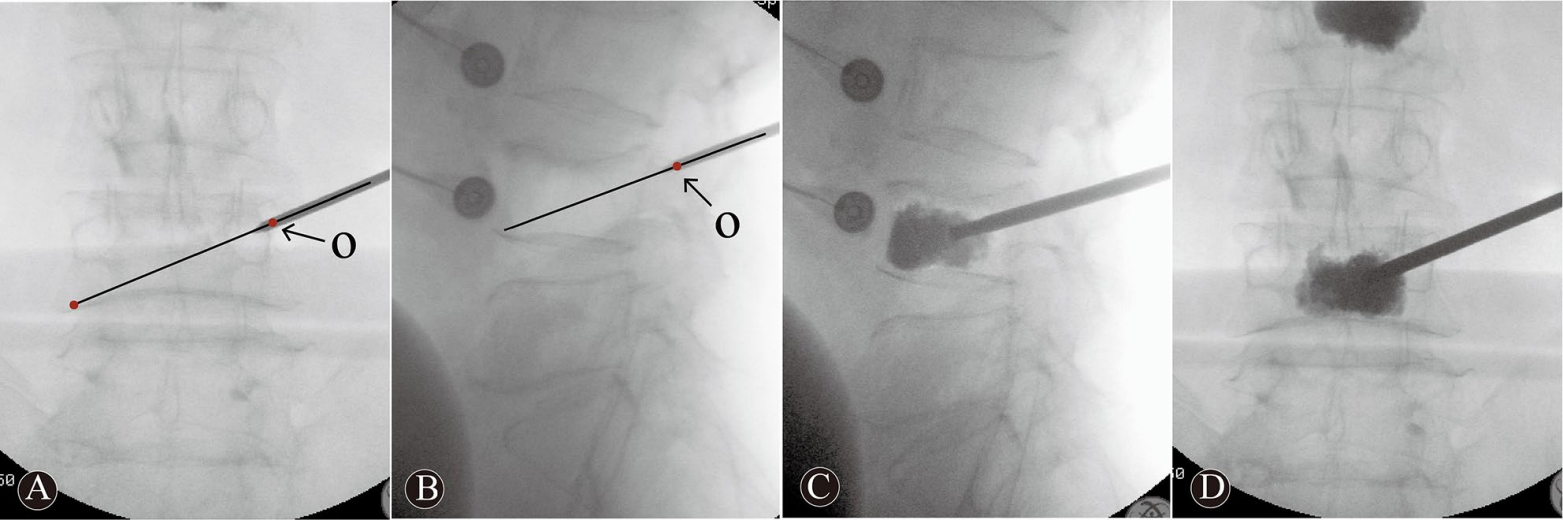
A 68 years-old woman in O-PKP group with L1, L2 and L4 OVCFs. These intraoperative images demonstrate operation techniques and procedures. The needle via point “O” to the contralateral inferior corner of vertebral body on posteroanterior imaging (**A**). The entry point “O” is located at the intersection of the posterior-superior margin of the pedicle and lateral margin to superior articular process on lateral imaging (**B**). These images (**C**, **D**) demonstrate an ideal bone cement distribution, the bone cement across the anterior one-third midline and across the midline of the vertebral body.

The O-PKP group: The O-PKP used a modified unilateral extrapedicular puncture approach. The bone entry point was the intersection between the base of the transverse process, the posterior-superior margin of the pedicle, and the lateral margin of the superior articular process. This entry point was defined as the “O” point (as this point was similar to the coordinate origin), as shown in Fig. 1A–C. Once the puncture needle reached the “O” point, it was close to the superior margin of the pedicle and was lateral to the superior articular process on lateral imaging (Fig. 2B). The puncture angle was adjusted such that the puncture needle entered the “O” point and the vertebral midpoint to the contralateral inferior corner of the vertebral body (Fig. 2A). The needle was then inserted slowly, using the anterior one-third midpoint of the vertebral body, as shown on lateral imaging, as the needle target. Posteroanterior imaging indicated that the needle had reached the vertebral midline. Lastly, a balloon was embedded in the vertebral body, and bone cement was then injected into the fractured vertebral body (Fig. 2C, D).

**Figure 3:**
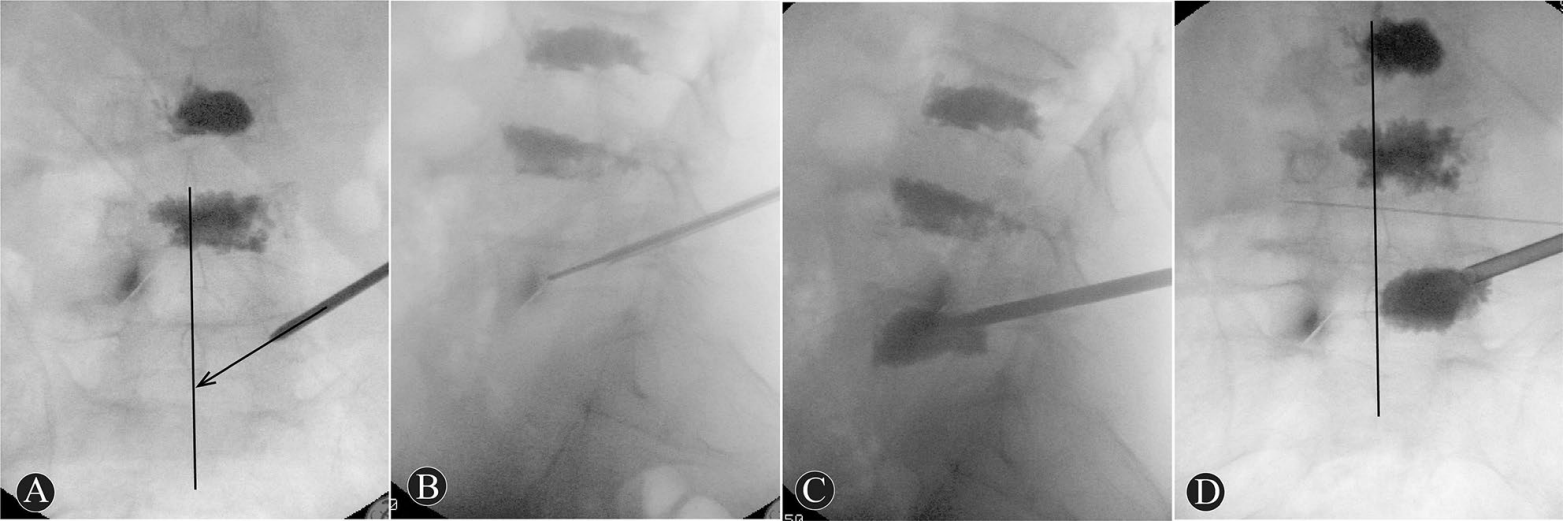
A 69 years old female patient in T-PKP group with L5 OVCF, previous PKP history of L3 and L4. These images are intraoperative X-ray fluoroscopy. The posteroanterior imaging (**A**) showed that the needle inclined to the midpoint of inferior endplates. The needle via the pedicle reached anterior 1/3 part of the vertebral body (**B**). The anteroposterior distribution of bone cement was satisfactory (**C**). Bone cement don’t across the midline of the vertebral body (**D**).

The T-PKP group: All patients in the T-PKP group underwent PKP via the transverse process-pedicle approach^9^. The bone entry point was located at the transverse process, and the entry point was below the “O” puncture point (Fig. 1A–B), 4–5 mm outside the lateral margin of the pedicle projection. The puncture needle reached the medial margin of the pedicle projection (Fig. 3A). Simultaneously, it was placed in the pedicle on the lateral imaging (Fig. 3B). The puncture angle was then adjusted to avoid the iliac crest. The range of the puncture angle was limited, which enabled inclination of the puncture needle in relation to the midpoint of the inferior endplates (Fig. 3A). The needle was then slowly inserted. The target point of the puncture needle was in the anterior one-third midpoint of the vertebral body on lateral imaging. However, as shown by posteroanterior imaging, the needle did not reach the midpoint of the vertebral body. At this point, a balloon was embedded into the vertebral body, and bone cement was injected into the fractured vertebral body (Fig. 3C, D).

No contralateral cement injections were administered to any of the patients in this study.

### Postoperative management

All patients were maintained with bed rest, and vital signs were monitored for a day after the operation. All patients received anti-osteoporosis medication after the operation, based on the guidelines for the management of osteoporosis and fragility fractures^11^. Daily activities, using sufficient brace protection, were begun from the first day after the operation, and functional exercise was started once the back pain was sufficiently relieved to not affect daily life. The postoperative hospital stay was 2–3 days.

### Date collection

The patients’ basic clinical information was collected and analyzed (Table 1). The operative time, blood loss, and fluoroscopy times were recorded. The operative time was defined as the time interval between anesthesia and sterile dressings. The intraoperative bleeding amount was measured using the weight increase of gauze (1 g equaled approximately 1 ml of lost blood). Bone cement leakage and volume were carefully recorded. Follow-up was conducted via outpatient visit and telephone. Owing to the COVID-19 outbreak, over half of the patients were followed up by telephone at the final follow-up. The VAS and ODI scores were assessed before the operation, on the first day after operation, and at the final follow-up. The VAS and ODI scores were used to evaluate the pain intensity and ability to conduct daily life activities, respectively.

**Table 1:** Comparison of patient baseline data between two groups.

Date	O-PKP (n = 31)	T-PKP (n = 31)	*P* value
Age(years)	73.00 ± 9.12	72.81 ± 8.97	0.935
**Sex**			0.530^#^
Male	11	8	
Female	20	23	
Course(days)	29.50(10.00 ~ 32.00)	10.00(6.25 ~ 55.50)	0.478*
**Trauma history**			0.250^#^
Yes	13	9	
No	18	22	
BMD(T-Score)	− 3.93 ± 1.03	− 4.08 ± 0.92	0.513
L4 OVCF	25/36	23/37	0.512^#^
L5 OVCF	11/36	14/37	
Other fracture	23/31	26/31	0.349^#^
HVF	2/31	3/31	1.000^#^
Follow-up time (months)	18.90 ± 6.25	16.06 ± 6.58	0.087

O-PKP, PKP via the entry point “O”; T-PKP, PKP via transverse process-pedicle approach; BMD, bone mineral density; HVF, Historic vertebral fracture. ^#^Data were analyzed by Chi-square test. *Data were analyzed by Friedman rank sum tests and data were expressed as medians (IQR).

**Figure 4:**
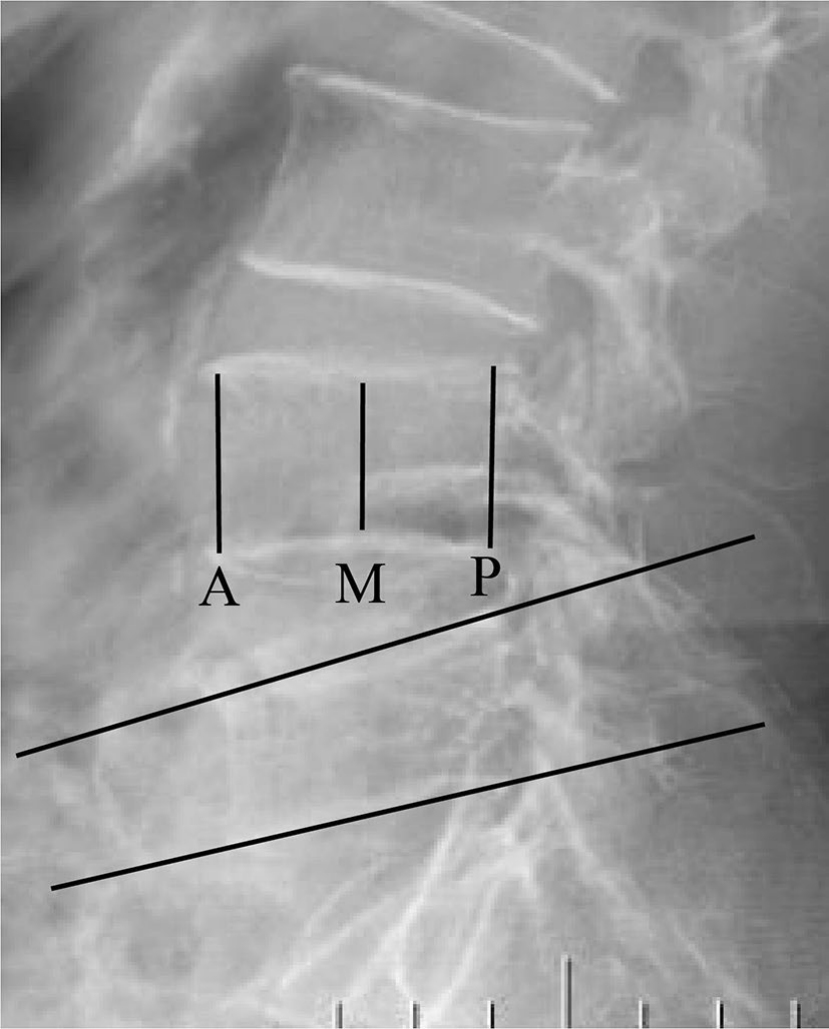
The local Cobb angle was measured to determine the kyphotic deformity on lateral radiographs. A, M and P represented anterior, middle and posterior height of vertebral body, respectively. The height of the fractured vertebral body was assessed via the compression ratio before and after operation, the compression ratio was computed by A/P $\times$ 100% and M/P $\times$ 100%.

Preoperative plain radiographs, three-dimensional reconstructed CT images, and magnetic resonance images were obtained for all patients. In addition, postoperative plain radiographs were also evaluated. The local Cobb angle was measured to determine the kyphotic deformity on lateral radiographs^12^. The anterior/middle/posterior height of the vertebral bodies (distance between the upper and lower endplates) was measured by the mean of the lateral radiographs (Fig. 4). The height of the fractured vertebral body was assessed as the vertebral compression ratio before and after the operation. The compression ratio was computed by the anterior vertebral height / posterior vertebral height (A/P)$\times$ 100%, and the middle vertebral height /posterior vertebral height (M/P)$\times$ 100%. The bilateral distribution on lateral imaging was considered satisfactory only when the bone cement was distributed evenly across the vertebral midline. It was also considered good when the anteroposterior distribution of the bone cement on preoperative imaging was well distributed across the anterior one-third midline of the vertebral body^13^. The sagittal and transverse angles were measured on three-dimensional reconstructed CT images (Fig. 1A-B).

### Statistical methods

SPSS version 25.0 was used for all data analyses. The normality of the quantitative data was assessed using the Kolmogorov–Smirnov method. Normally distributed data of the inter- and subgroup populations were analyzed by t-tests, while intergroup differences were analyzed by the Bonferroni method. The intergroup differences in bone cement distribution were assessed by χ^2^ tests. Non-normally distributed data were analyzed using the Friedman rank sum test. Non-normally distributed data are presented as medians (interquartile range, IQR), while normally distributed data are given as means ± SD ($\overline{x }$ ± s). *P* < 0.05 was considered statistically significant.

## Results

### Patient baseline characteristics

No significant differences in sex, age, bone mineral density, and disease course, amongst other factors, were observed between the two groups (*P* > 0.05) (Table 1).

The O-PKP group included twenty women and eleven men, with a mean age of 73.00 ± 9.12 years old (ranging from 55 to 89 years old), a mean BMD (T-Score) of -3.93 ± 1.03 (ranging from − 6.2 to − 2.5), and a median disease course of 29.50 days (IQR, 10.00‒32.00). Eighteen patients did not have any traceable trauma history, while thirteen patients had a definite trauma history. Twenty-three patients experienced other vertebral fractures, with two patients having historic vertebral fractures. Lastly, there were 25 L4 OVCFs and 11 L5 OVCFs.

The T-PKP group included twenty-three women and eight male patients, with a mean age of 72.81 ± 8.97 years old (ranging from 40 to 88 years old), a mean BMD (T-Score) of − 4.08 ± 0.92 (ranging from − 5.9 to − 2.5), and a median disease course of 10.00 days (IQR, 6.25‒55.50). Twenty-two cases did not report any traceable trauma history, while nine cases had a definite trauma history. Twenty-six patients experienced additional vertebral fractures, and three patients had historic vertebral fractures. Lastly, there were 23 L4 OVCFs and 14 L5 OVCFs.

### Surgical outcome

The surgery was carried out, without any major complications, in all patients, and they were discharged uneventfully. The operative time in the O-PKP and T-PKP groups were 20.42 ± 3.48 min and 23.08 ± 3.21 min, respectively. The blood loss in the O-PKP and T-PKP groups were 10.80 ± 2.20 ml and 11.42 ± 2.08 ml, respectively. The fluoroscopy times in the O-PKP and T-PKP groups were 20.97 ± 3.21 and 22.70 ± 3.08, respectively (Table 4). The operative time and fluoroscopy times in the O-PKP group were significantly less than the T-PKP group (*P* < 0.05), and there was no significant difference in the blood loss between the two groups (*P* > *0.05*). The average volume of cement in O-PKP group and T-PKP group was 5.56 ± 0.47 ml and 5.42 ± 0.52 ml, respectively, and there was no significant difference between the two groups (Table 4). There was no bone cement leakage in the O-PKP group. However, intervertebral disc space leakage occurred in two cases in the T-PKP group. Notably, there was no evidence of bone cement intraspinal leakage.

### Follow-up outcomes

#### Clinical outcomes

The O-PKP and T-PKP group follow-ups were at 18.90 ± 6.25 and 16.06 ± 6.58 months, respectively (Table 1).

The O-PKP group: The median VAS score on the first day post-operation and at the final follow-up were significantly decreased from the preoperative score of 6.0 (IQR, 6.0‒7.0) to the post-operative score of 2.0 (IQR, 2.0‒3.0), and 1.0 (IQR, 1.0‒1.0), respectively. The median ODI on the first day post-operation and at the final follow-up were significantly reduced from preoperative 72.0 (IQR, 71.0‒73.0)% to post-operative scores of 24.0 (IQR, 22.3‒25.8)% and 10.5 (IQR, 9.0‒11.0)%, respectively.

The T-PKP group: The median VAS scores on the first day post-operation and at the final follow-up were significantly decreased from the preoperative score of 6.0 (IQR, 6.0‒7.0) to the postoperative score of 2.0 (IQR, 2.0–3.0) and 2.0 (IQR, 2.0‒3.0), respectively. The median ODI on the first day post-operation and at the final follow-up were significantly decreased from the preoperative score of 72.0 (IQR, 71.0‒73.0)% to the postoperative scores of 25.0 (IQR, 24.0‒26.0)% and 12.0 (IQR, 11.0‒13.0)%, respectively.

Thus, compared with the preoperative scores, there were significant reductions in the postoperative VAS and ODI scores in both groups. However, the VAS and ODI scores in the O-PKP group were far better than those in the T-PKP group at the final follow-up (*P* < 0.05) (Table 2). Notably, one patient suffered adjacent vertebral fracture within 1 month after the operation in the O-PKP group.

**Table 2:** VAS and ODI scores were evaluated at pre-operation, post-operation 1 day and the final follow-up between groups.

Parameter	O-PKP	T-PKP	*P* value
**Pre-operation**			
VAS	6.0 (6.0 ~ 7.0)	6.0 (6.0 ~ 7.0)	0.962
ODI (%)	72.0 (71.0 ~ 73.0)	72.0 (71.0 ~ 73.0)	0.696
**Post-operation 24 h**			
VAS	2.0 (2.0 ~ 3.0)	2.0 (2.0 ~ 3.0)	0.604
ODI (%)	24.0 (22.3 ~ 25.8)	25.0 (24.0 ~ 26.0)	0.044
**The final follow-up**			
VAS	1.0 (1.0 ~ 1.0)	2.0 (2.0 ~ 3.0)	< 0.001
ODI (%)	10.5 (9.0 ~ 11.0)	12.0 (11.0 ~ 13.0)	< 0.001

The VAS and ODI scores were analyzed using the Friedman rank sum tests, due to these data did not conform to a normal distribution, these data expressed as medians (IQR).

#### Radiographic outcomes

The postoperative anterior and middle vertebral heights and the local Cobb angle were markedly improved in both groups compared with the preoperative values (*P* < 0.05) (Table 3). However, the increased vertebral compression ratio in the O-PKP group was significantly better than that in the T-PKP group (*P* < 0.05) (Table 4). Interestingly, there was no significant difference in the Cobb angle change between the two groups (*P* > 0.05) (Table 4). The anteroposterior distribution of the bone cement was satisfactory, with no significant difference between the two groups (*P* > 0.05). The bilateral distribution of the bone cement in the O-PKP group was better than that in the T-PKP group (*P* < 0.05) (Table 4).

**Table 3:** The height of vertebral body and Cobb angle were evaluated at pre-operation and post-operation.

Parameter	O-PKP				T-PKP			
	Pre-operation	Post-operation	*t*	*P* value	Pre-operation	Post-operation	*t*	*P* value
AVH (cm)	1.79 ± 0.33	2.13 ± 0.31	− 4.4866	< 0.001	1.82 ± 0.30	2.04 ± 0.27	− 3.2023	0.002
MVH (cm)	1.63 ± 0.49	1.89 ± 0.44	− 2.4197	0.018	1.86 ± 0.43	2.05 ± 0.35	− 2.0262	0.046
PVH (cm)	2.32 ± 0.40	2.39 ± 0.37	− 0.7009	0.486	2.33 ± 0.27	2.41 ± 0.25	− 1.2362	0.220
Cobb (°)	14.39 ± 4.47	6.73 ± 2.62	14.7001	< 0.001	15.11 ± 3.94	6.74 ± 2.47	16.9501	< 0.001

AVH, anterior vertebral height; MVH, middle vertebral height; PVH, posterior vertebral height. The data were analyzed by t-test.

**Table 4:** Comparisons of clinical and radiographic parameters between groups.

Parameter	O-PKP	T-PKP	t (*x*^2^)	*P* value
Operative time (min)	20.42 ± 3.48	23.08 ± 3.21	− 3.4044	0.001
Blood loss(ml)	10.80 ± 2.20	11.42 ± 2.08	− 1.2383	0.220
Fluoroscopy times	20.97 ± 3.21	22.70 ± 3.08	− 2.3496	0.022
Cement volume(ml)	5.56 ± 0.47	5.42 ± 0.52	1.9303	0.058
L4 sagittal angler (°)	31.97 ± 6.71	21.37 ± 8.23	4.9087	< 0.001
L4 transverse angle (°)	38.62 ± 2.77	33.97 ± 3.03	5.5622	< 0.001
L5 sagittal angler (°)	45.45 ± 5.67	33.46 ± 6.72	4.7300	< 0.001
L5 transverse angle (°)	50.79 ± 4.43	38.94 ± 2.40	8.5665	< 0.001
**Pre-operation**				
AVH (cm)	1.79 ± 0.33	1.82 ± 0.30	− 0.4832	0.630
MVH (cm)	1.74 ± 0.37	1.84 ± 0.40	− 1.0542	0.295
PVH (cm)	2.33 ± 0.40	2.33 ± 0.27	− 0.0515	0.959
A/P %	77.23 ± 9.12	78.02 ± 7.86	− 0.3948	0.694
M/P %	75.34 ± 12.52	78.53 ± 13.42	− 1.0522	0.296
**Post-operation**				
AVH (cm)	2.13 ± 0.31	2.04 ± 0.27	1.3230	0.190
MVH (cm)	2.07 ± 0.32	2.03 ± 0.26	0.6318	0.530
PVH (cm)	2.39 ± 0.37	2.39 ± 0.23	− 0.1110	0.913
A/P (%)	89.71 ± 8.78	85.09 ± 8.00	2.3525	0.021
M/P (%)	87.13 ± 7.54	84.68 ± 7.23	1.4116	0.162
△A/P (%)	12.48 ± 6.74	7.07 ± 6.24	3.5598	0.001
△M/P (%)	11.79 ± 8.67	6.15 ± 10.98	2.4319	0.018
△Cobb(°)	− 7.66 ± 3.12	− 8.36 ± 3.00	0.9856	0.328
**BCD**				
Right-left	34	28	5.022	0.046*
Anterior–posterior	36	35	2.001	0.493*

A/P, anterior vertebral height / posterior vertebral height $\times$ 100%; M/P, middle vertebral height /posterior vertebral height $\times$ 100%; △A/P = postoperative A/P—preoperative A/P; △M/P = postoperative M/P—preoperative M/P; △Cobb = postoperative Cobb—preoperative Cobb; BCD, bone cement distribution. * With theoretical cases less than 5 in a cell, Fisher's exact test method was used.

The L4 and L5 sagittal angles in the O-PKP were 31.97 ± 6.71° and 45.45 ± 5.67°, respectively. The L4 and L5 transverse angles in the O-PKP group were 38.62 ± 2.77° and 50.79 ± 4.43°, respectively. The L4 and L5 sagittal angles in the T-PKP group were 21.37 ± 8.23° and 33.46 ± 6.72°, respectively. The L4 and L5 transverse angles in the T-PKP group were 33.97 ± 3.03° and 38.94 ± 2.40°, respectively. The sagittal and transverse angles in the O-PKP group were substantially larger than in the T-PKP group (*P* < 0.05) (Table 4), and the sagittal and transverse angles of L5 were larger than L4 (*P* < 0.05).

## Discussion

PKP is a widely used procedure^14^. Common unilateral puncture approaches include CPA, TPA, and the extrapedicular approach. However, the anatomical characteristics of the L4 and L5 vertebrae are quite different, as the length/ width ratio of the lumbar vertebral bodies decreases from L1 to L5 with the L5 vertebral body being the widest. The width of the pedicle gradually widens from L1 to L5, and the transverse angle of the pedicle gradually increases from L1 to L5^15^. In addition, in terms of ileac shielding, it is often difficult for traditional puncture approaches to reach the contralateral side of the vertebral body using a unilateral approach^6,9^. In this study, combined with the anatomical characteristics of the L4 and L5 vertebrae, we demonstrated that the intersection of the base of the transverse process, the posterior-superior margin of the pedicle, and the lateral margin to the superior articular process was an ideal anatomical position for the entry point into the bone surface. PKP via this entry point, termed “O”, is thus a modified extrapedicular approach that circumvents the challenges faced by other procedures.

The entry point “O” is located at the superior margin of the pedicle and, therefore, the use of this trajectory avoids injury to the exiting nerve root below the infravertebral notch^6^. In addition, this also results in a larger sagittal angle, relative to the CPA and TPA approaches. This larger sagittal incline facilitates an easier puncture approach while avoiding iliac shielding. Compared to CPA and TPA, the “O” entry point is closer and more lateral to the vertebral body, resulting in a larger and safer transverse angle range. In particular, this trajectory allows easier access to the contralateral side of the L4 or L5 vertebral body using a unilateral approach, especially with a reduced risk of breaking the inner pedicle wall. The “O” entry point is also distant from the peripheral nerves and blood vessels^16,17^, and is thus located in a safe area of operation^15^. In addition, the “O” entry point can be clearly visualized on both CT and X-ray imaging.

An elevated ilium has a significant influence on lower lumbar surgery, particularly at L5. Zhang et al. reported that the iliac height produces greater occlusion than the width at the L5 pedicle screw^18^. In addition, the trajectory of the percutaneous endoscopic discectomy can be shielded by the ilium at the L4-S1, particularly at the L5-S1^19^. Tezuka reported that an approach using a larger head dip can be employed to overcome iliac shielding^19^. In this study, the sagittal angle was larger in the O-PKP group than in the T-PKP group. Therefore, the puncture approach could easily avoid iliac shielding and the bone cement was able easily to fill the area around the upper and lower endplates of the vertebral body. Several studies have shown that the risk of vertebral refracture is lowest when the distribution of bone cement is located near the upper and lower endplates^20,21^. Moreover, the operative time and fluoroscopy times in the O-PKP group were significantly less than in the T-PKP group, which is consistent with a study by Ge et al.^22^. One potential explanation for these excellent intraoperative parameters in the O-PKP group was that the entry point “O” was readily observed on C-arm fluoroscopy, and without iliac shielding, less time was needed for adjustment and fluoroscopy of the puncture needle.

Our post-operative follow-ups showed continuous improvement in symptoms without any serious complications. Only one case in the O-PKP group required prolonged glucocorticoid administration, and the patient suffered an adjacent vertebral fracture within 1 month of the procedure. Continuous glucocorticoid intake can impair the dynamic balance between bone formation and bone resorption, thereby accelerating bone loss while increasing the fracture risk^23^. Compared to the preoperative scores, the postoperative VAS and ODI scores in the two groups were significantly decreased (*P* < 0.05). However, the VAS and ODI scores in the O-PKP group were considerably better than those in the T-PKP group at the final follow-up (*P* < 0.05). Ge et al.^24^ reported a mean VAS score of 2.16 ± 0.57 at 3 days post-operation after unilateral extrapedicular puncture. This result is consistent with the results of our study. However, the VAS scores in the O-PKP group were less than those reported by Ge et al.^22^ at the final follow-up. The possible reason why the clinical outcomes in the T-PKP group were worse than the O-PKP group may be an asymmetric distribution of the bone cement. Some researchers have speculated that insufficient bone cement distribution is associated with reduced pain relief and kyphotic deformity^21,25^. Liebschner et al.^20^ reported that asymmetric bone cement distribution leads to height loss in the contralateral side of the vertebral body with less cement. In this study, we demonstrated that the transverse angle in the O-PKP group was larger than in the T-PKP group, making it easier to access the contralateral side of the L4 or L5 vertebral body using a unilateral approach in the O-PKP group. This may explain why the bone cement distribution and vertebral reconstruction in the O-PKP group showed obvious advantages over the T-PKP group (*P* < 0.05).

In this study, we did not observe any leakage of bone cement in the O-PKP group, while two cases of bone cement leakage in the intervertebral disc space occurred in the T-PKP group. Fortunately, there was no evidence of intraspinal leakage of bone cement in either of the groups, which may be due to the integrity of the posterior wall of the vertebral body and inner wall of the pedicle. In addition, bone cement leakage is associated with both the volume and viscosity of the bone cement^26^. In this study, there was no significant difference in bone cement volunme between the two groups. The larger the bone cement volume, the higher the risk of cement leakage^27^. Several researchers have speculated that when the bone cement volume ratio of the punctured side exceeds the optimal ratio of 13.68%, there is a significant increase in bone cement leakage in the paravertebral vein^28^. To reduce the risk of cement leakage, the optimal volume of low-viscosity bone cement requires reduction^29^. Based on our clinical experience, it is better to first inject low-viscosity bone cement and then administer high-viscosity bone cement. This approach is not only advantageous for bone cement diffusion but also minimizes bone cement leakage.

## Conclusion

Based on our analyses, O-PKP was highly beneficial for patients with L4 or L5 OVCF. This puncture approach exhibited significant advantages not only in pain relief, operative time, and fluoroscopy times, but also in the puncture angle, vertebral reconstruction, and symmetric bone cement distribution. The number of cases in this study was relatively small, and the follow-up time post-operation was also relatively short. The study lacked the prospective and multi-center control research approach. Therefore, some clinical and radiographic outcomes may not be observed. Our future studies will further investigate imaging of the anatomical feasibility of O-PKP.

## Data Availability

The raw data required to reproduce these findings cannot be shared at this time as the data also forms part of an ongoing study, but are available from the corresponding author on reasonable request.
